# Genetic Variation in *BCL2* 3′-UTR Was Associated with Lung Cancer Risk and Prognosis in Male Chinese Population

**DOI:** 10.1371/journal.pone.0072197

**Published:** 2013-08-16

**Authors:** Ping Xu, Li Liu, Jianzhong Wang, Kai Zhang, Xiaohua Hong, Qifei Deng, Jingjun Xiang, Xiaomin Zhang, Meian He, Tangchun Wu, Huan Guo

**Affiliations:** 1 Department of Occupational and Environmental Health and Ministry of Education Key Lab for Environment and Health, School of Public Health, Tongji Medical College, Huazhong University of Science and Technology, Wuhan, China; 2 Department of Oncology, Wuhan Iron and Steel (Group) Corporation Staff-Worker Hospital, Wuhan, China; 3 Department of Oncology, Cancer Center, Union Hospital, Huazhong University of Science and Technology, Wuhan, China; Kyushu University Faculty of Medical Science, Japan

## Abstract

**Objectives:**

Bcl-2 is a critical apoptosis inhibitor with established carcinogenic potential, and can confer cancer cell resistance to therapeutic treatments by activating anti-apoptotic cellular defense. We hypothesized that genetic variants of *BCL2* gene may be associated with lung cancer susceptibility and prognosis.

**Methods:**

Three selected tagSNPs of *BCL2* (rs2279115, rs1801018, and rs1564483) were genotyped in 1017 paired male Chinese lung cancer cases and controls by TaqMan assay. The associations of these variants with risk of lung cancer and overall survival of 242 male advanced non-small-cell lung cancer (NSCLC) patients were separately investigated.

**Results:**

Compared with the *BCL2* 3′UTR rs1564483GG genotype, the rs1564483GA, AA, and GA+AA genotypes were associated with significantly decreased susceptibilities of lung cancer in male Chinese (adjusted OR = 0.78, 0.73, and 0.76, *P* = 0.016, 0.038, and 0.007, respectively), while rs1564483A allele has a inverse dose-response relationship with lung cancer risk (*P*_trend_ = 0.010). These effects were more evident in the elders, smokers, and subjects without family history of cancer (*P*_trend_ = 0.017, 0.043 and 0.005, respectively). Furthermore, advanced NSCLC males carrying *BCL2* rs1564483 GA+AA genotypes had significantly longer median survival time (Long-rank *P* = 0.036) and decreased death risk (adjusted HR = 0.69, *P* = 0.027) than patients with rs1564483GG genotype. These effects were more obvious in patients with smoking, stage IIIA, and in patients without surgery but underwent chemotherapy or radiotherapy (adjusted HR = 0.68, 0.49, 0.67, 0.69, 0.50, respectively, all *P*<0.05).

**Conclusion:**

The *BCL2* 3′UTR rs1564483A allele was associated with a decreased lung cancer risk and better survival for advanced NSCLC in male Chinese, which may offer a novel biomarker for identifying high-risk population and predicting clinical outcomes.

## Introduction

Lung cancer is the most common malignancy with leading cause of cancer-related mortality in China and throughout the world [1]. Approximately 80% lung cancer patients were non-small cell lung cancer (NSCLC), and the majority of NSCLC patients were diagnosed at an advanced stage [2]. Although more than 85% of lung cancer death are attributed to cigarette smoking [3], only a fraction of smokers finally develop lung cancer, which highlights the potential role of genetic susceptibility in this disease. For more than two decades, the most effective systemic chemotherapy for advanced NSCLC was platinum-based combination treatment, but the effectiveness has apparently reached a plateau, with the overall 5-year survival rate of still only 15% [1]. Identifying special genetic biomarkers to guide personalized therapy strategy was crucial for minimizing therapy resistance and may improve the clinical outcome of patients with NSCLC.

Apoptosis has now been widely accepted as a prominent suppression mechanism of lung cancer, and it can be activated through an intrinsic Bcl-2 pathway and an extrinsic death receptor pathway [4]. Pro-apoptotic and anti-apoptotic members of the Bcl-2 family control crucial checkpoints of mitochondrion-initiated intrinsic apoptotic pathway, where Bcl-2 acts as a critical anti-apoptotic regulator [5]. The oncogenic potential of Bcl-2 has been well established [6]. It can inhibit apoptosis from various stress stimuli, such as DNA damage, microtubule perturbation, and oncogene activation [7]. In addition, Bcl-2 has been reported to prevent the intrinsic apoptotic pathway through interaction with a variety of pro-apoptotic factors and suppression the release of cytochrome *c* from mitochondria through anion channels, the formation of apoptosome, and the subsequent activation of the cascade of effector caspases. [5], [8], [9]. It has also been demonstrated that, beyond roles in carcinogenesis, Bcl-2 can activate anti-apoptotic cellular defense of lung cancer cells to therapeutic treatments, such as cytotoxic chemotherapy, radiotherapy, and monoclonal antibodies, which may affect the prognosis of lung cancer patients [10]. Various single-nucleotide polymorphisms (SNP) in apoptotic genes have been demonstrated to contribute to lung cancer [11]–[13], but data is scare on the associations of *BCL2* variants with lung cancer risk and prognosis.

Since Bcl-2 may play a crucial role in regulating apoptosis and survival of both normal and malignant lung cells, the aim of this study is to investigate whether the genetic variations of *BCL2* gene with were associated the susceptibility and survival outcome of lung cancer in male Chinese.

## Materials and Methods

### Ethics Statement

The study subjects provided their written informed consent after a clear explanation of study objective. All subjects are genetically unrelated ethnic Han Chinese and this study is approved by the Institutional Review Board of Tongji Medical College, Huazhong University of Science and Technology.

### Study Population

The study population of this case-control study had been previously mentioned [14]. Briefly, we recruited 1017 male lung cancer cases from the Union Hospital Cancer Center, Wuhan Steel Group/Corporation Staff-Worker Hospital and Wuhan Zhongnan Hospital between January-2003 and December-2009 in Wuhan City, Hubei Province of Central China. To do a 1∶1 frequency match to these lung cancer cases on age (±5 years) and sex, we randomly selected 1017 male healthy subjects from a heath examination of 4073 individuals in the same City during the same period.

In the case-only survival cohort, we followed up the patients enrolled in Wuhan Iron and Steel Group/Corporation Staff-Worker Hospital between January-2003 to December-2009, because patients at this hospital were employees of Wuhan Steel Group/Corporation, who lived in the same region and had a similar socio-economic status. After being diagnosed with lung cancer, these patients received treatment at the same hospital until they died from the disease, and more than 98% patients keep good follow-up. In order to minimize the bias due to patient selection, inconsistency of therapies, and individual socio-economic status among patients in different hospitals, the 242 male advanced NSCLC patients who had completed follow-up and clinical information from Wuhan Iron and Steel Group/Corporation Staff-Worker Hospital were included in the survival analysis. All these male NSCLC patients were included in the 1017 cases of above case-control study. A large part of the subjects has been published [14]. The TNM stage classification was evaluated by medical oncologists according to the Staging Manual of AJCC/UICC [15]. Patients were followed up by telephone calls every three months until December 31, 2010. Date of death was obtained from inpatient and outpatient records or patients’ families through follow-up telephone calls. Patients who were still alive on December 31, 2010 were considered as censored, and the survival time for each patient was calculated from the date when patients were confirmed diagnosed of lung cancer until the date of death or the last follow-up.

All patients and control subjects provided their written informed consent to participate in the study. Information on demographic characteristics, smoking habits, alcohol consumption, medical history, and family history of cancer were collected via an interviewing using a pretested questionnaire. Those who had smoked less than one cigarette per day for less than one year over their entire lifetime were defined as non-smokers; those who had stopped smoking for more than 1 year previously were considered former smokers; and those who were still smoking in the previous year were defined as current smokers.

### SNP Selection and Genotyping

Genomic DNA was extracted using the Gentra puregene blood kit (Qiagen, Hilden, Germany) following the manufacturer’s instructions. The three *BCL2* polymorphisms, rs2279115 C>A (−938C>A), rs1801018A>G (+21A>G), and rs1564483G>A (c.*1204G>A), were the most frequently studied SNPs located in the functional region of the 5′-promoter, exon-2, and 3′- untranslated region (UTR) of *BCL2* gene, respectively. In this study, the genotyping of these three *BCL2* polymorphisms in all subjects was carried out by the TaqMan® method using the ABI 7900HT Sequence Detection System (Applied Biosystems). All primers and probes were ordered from Applied Biosystems. For the rs2279115 C>A polymorphism, the TaqMan primers were 5′- GCATTTGCTGTTCGGAGTTT -3′ and 5′- ATCCACGGGACCGCTTCAC -3′, while probes were FAM- TTCATCGTCCCCTCTCCCCTGTC -MGB for rs2279115C and VIC- CTTCATCGTCCCATCTCCCCTGTCT -MGB for rs2279115A. The catalog numbers for the rs1801018 A>G and rs1564483 G>A polymorphisms were C_11449823_10 and C_7905447_1_, respectively. The cycling conditions were as follows: 50°C for 2 minutes, initial denaturation at 95°C for 10 minutes, and followed by 45 cycles consisting of 95°C for 15 seconds and 60°C for 1 minute.

### Statistical Analysis

The one-sample Komogorov-Smirnov normality test was used to evaluate the distributions of continuous variables, and the Chi-square test was used to compare the distributions of categorical variables between case and control subjects and to calculate Hardy-Weinberg equilibrium of each variant in the control group. In all subjects and in the stratified subgroups, the multiple logistic regression analyses were conduct to evaluate the associations of each SNP with the risk of lung cancer, adjusting for age, smoking status, pack-years and family history of cancer. The effect modifications by age, smoking status, pack-years smoked, family history of cancer and SNP on lung cancer risk were also tested in the multiple logistic regression models. The Kaplan-Meier method and log-rank test were used to calculate and compare the median survival time (MST) of patients with different *BCL2* genotypes. The associations between *BCL2* SNPs and death risk of advanced NSCLC patients were estimated using the multivariate Cox regression models, with adjustment of age, smoking status, histology, TNM stage, and therapy treatments of surgical resection, chemotherapy, and radiotherapy. The effect modifications by age, stage and SNPs on death risk of male advanced NSCLC patients were assessed by using the Wald test in the multivariate Cox proportional hazards regression models after adjusting for the confounders. The construction of *BCL2* haplotypes and their associations with risk of lung cancer and risk of death among the advanced NSCLC patients were determined using THESIAS v3.1 software with adjustment for the above confounders, respectively. All analyses were conducted on the SPSS 13.0 software (SPSS Inc., Chicago, IL) and the two-tailed *P*<0.05 was considered statistically significant.

## Results

### BCL2 SNPs and Lung Cancer Risk

The general characteristics for the 1017 pairs of male lung cancer cases and control were showed in Table 1. The distributions of age were not different between case and control subjects, with the mean ages of 60.3±10.7 and 59.7±12.1, respectively. There were more smokers with higher smoking pack-years in the lung cancer cases than in the controls (*P*<0.001), and there were also more individuals with family history of cancer in cases than in controls (*P*<0.001). When comparing with the *BCL2* rs1564483 GG genotype, subjects with the rs1564483 GA or AA had significantly decreased risk of lung cancer (OR = 0.78 and 0.73, *P* = 0.016 and 0.038, respectively). When combining the rs1564483 GA and AA genotypes, we still found a strong decreased risk of lung cancer compared with GG genotype (OR = 0.76 and *P* = 0.007). There was a dose–response association between the increasing number of the rs1564483 A allele in decreased lung cancer risk (*P*_trend = _0.010). However, there were no significant associations of *BCL2* rs2279115 C>A and rs1801018 A>G with the risk of lung cancer in male Chinese (Table 2).

**Table 1:** General information of case patients and control subjects.

Variable	Cases, n (%)	Controls, n (%)	*P* *
	N = 1,017	N = 1,017	
Age (years)			
Mean ± SD	60.3±10.7	59.7±12.1	0.179
≤50	181(17.8)	192(18.9)	0.341
51∼60	321(31.6)	287(28.2)	
61∼70	324(31.9)	351(34.5)	
>70	191(18.8)	187(18.4)	
Smoking status			<0.001
Never	151(14.8)	356(35.0)	
Former	370(36.4)	140(13.8)	
Current	496(48.8)	521(51.2)	
Pack-years smoked			<0.001
0	151(14.8)	356(35.0)	
≤26	215(21.1)	326(32.1)	
>26	651(64.0)	335(32.9)	
Family history of cancer			<0.001
No	886(87.2)	969(95.4)	
Yes	130(12.8)	48(4.7)	
Histological type			
Adenocarcinoma	276(27.3)		
Squamous cell carcinoma	365(36.1)		
Small cell carcinoma	48(4.7)		
Others†	323(31.9)		

* Two-sided chi-square test.

† Others include large cell, bronchioalveolar, mixed cell, undifferentiated and pathologic not otherwise specified carcinomas.

**Table 2:** Genotype frequencies of *BCL2* among case and control subjects and their associations with risk of lung cancer.

Genotypes	Cases, n (%)	Controls, n (%)	OR(95% CI)*	*P* *
*BCL2_rs2279115*				
−938C/A				
CC	378(37.2)	393(38.6)	Reference	–
CA	483(47.5)	479(47.1)	1.02(0.83–1.25)	0.862
AA	156(15.3)	145(14.3)	1.11(0.84–1.47)	0.474
*BCL2_rs1801018*				
+21A>G				
AA	855(84.1)	846(83.2)	Reference	–
AG	152(14.9)	159(15.6)	0.92(0.71–1.19)	0.513
GG	10(1.0)	12(1.2)	0.95(0.39–2.33)	0.916
AG+GG	162(15.9)	171(16.8)	0.92(0.72–1.18)	0.513
*BCL2_rs1564483*				
c*1204G>A				
GG	433(42.6)	368(36.2)	Reference	
GA	461(45.3)	506(49.8)	0.78(0.64–0.95)	0.016
AA	123(12.1)	143(14.1)	0.73(0.54–0.98)	0.038
GA+AA	584(57.4)	649(63.8)	0.76(0.63–0.93)	0.007
			*P*_trend_ *	0.010
*BCL2_*Haplotypes†				
C-A-G	817(40.2)	763(37.5)	Reference	–
C-A-A	422(20.7)	501(24.6)	0.81(0.67–0.98)	0.028
A-A-G	406(20.0)	378(18.6)	1.02(0.83–1.25)	0.847
A-A-A	217(10.7)	210(10.3)	0.98(0.76–1.27)	0.879

Note: Associations were determined for haplotypes >5% frequency.

* Data were calculated by unconditional logistic regression models, adjusted for age, smoking status, pack-year smoked, and family history of cancer.

† Polymorphic bases were in the order of rs2279115 C>A, rs1801018 A>G, rs1564483 G>A from 5′ to 3′. Frequencies of haplotypes were determined using THESIAS v3.1 software.

### BCL2 Haplotypes and Lung Cancer Risk

Haplotype may be a real representation of the combinatorial appearance of all genetic variations. We further constructed the *BCL2* haplotypes and assessed their associations with lung cancer risk by using the THESIAS v3.1 software, after adjustment of age, smoking status, pack-years smoked, and family history of cancer. It is shown in Table 2 that when compared to the major CAG haplotype, the CAA haplotype was associated with a significantly decreased risk of lung cancer (adjusted OR = 0.81, *P* = 0.028), while no significant associations were shown for the AAG and AAA haplotypes.

### Stratification Analysis for Associations between BCL2 rs1564483 and Lung Cancer Risk

We further evaluated the associations between *BCL2* rs1564483 genotypes and risk of NSCLC stratified by subgroups of age, smoking status, pack-years smoked, and histological type, assuming both additive and dominant genetic models based on above results. It was shown that the association of rs1564483 GA+AA genotype with decreased susceptibility of lung cancer was more robust in the elders (age >60) (OR = 0.67, *P = *0.004), smokers (OR = 0.81, *P* = 0.049), and subjects without family history of cancer (OR = 0.73, *P = *0.003) (Table 3). There is an evident dose–response effect of rs1564483 A allele in reducing lung cancer risk in above subgroups (*P*_trend_ = 0.017, 0.043, 0.044, and 0.005, respectively). In addition, age can significantly modify the effect of rs1564483A allele in decreasing lung cancer risk (*P*_interaction_ = 0.008), but no significant interactions were found between rs1564483 A allele and smoking, pack-years smoked, or family history of cancer on the susceptibility of lung cancer (*P*_interaction_ = 0.659, 0.931, and 0.124, respectively).

**Table 3:** Stratification analysis for associations between *BCL2* rs1564483 (c*1204G>A) genotypes and risk of lung cancer.

Variables	GG	GA		AA		GA+AA		*P*_trend_	*P*_interaction_
	OR	OR(95%CI)	*P*	OR(95%CI)	*P*	OR(95%CI)	*P*		
Age									0.008
≤60	1.00	0.91(0.68–1.21)	0.519	0.77(0.50–1.17)	0.214	0.88(0.67–1.15)	0.343	0.220	
>60	1.00	0.66(0.49–0.88)	0.005	0.71(0.46–1.09)	0.116	0.67(0.51–0.88)	0.004	0.017	
Smoking status									0.659
Never-smokers	1.00	0.72(0.48–1.10)	0.126	0.75(0.40–1.41)	0.375	0.73(0.49–1.08)	0.117	0.188	
Smokers	1.00	0.83(0.67–1.04)	0.109	0.75(0.54–1.03)	0.076	0.81(0.66–1.00)	0.049	0.043	
Pack-years smoked									0.913
≤26	1.00	0.79(0.54–1.15)	0.223	0.90(0.53–1.53)	0.704	0.82(0.57–1.16)	0.263	0.452	
>26	1.00	0.81(0.61–1.08)	0.152	0.67(0.44–1.02)	0.062	0.78(0.59–1.02)	0.073	0.044	
Family history of cancer									0.124
No	1.00	0.74(0.60–0.92)	0.006	0.71(0.52–0.97)	0.030	0.73(0.60–0.90)	0.003	0.005	
Yes	1.00	1.34(0.62–2.90)	0.453	0.96(0.29–3.27)	0.959	1.26(0.61–2.61)	0.534	0.743	

Note: ORs and *P* values were obtained from logistic regression models with adjustment for age, smoking status, pack-year smoked, and family history of cancer.

### BCL2 SNPs and Survival of Male Advanced NSCLC Patients

In the survival cohort of male advanced NSCLC patients, only 3 male patients were lost to follow-up. Two persons carry the rs1564483GG genotype and one person carries the rs1564483AA genotype. So we do not include these patients in the further survival analysis. The demographic and clinical characteristics of the 242 male advanced NSCLC patients who had completed follow-up information are listed in Table 4. In these patients, the mean age of was 64.17±9.36 years, 185 (76.4%) patients died of lung cancer, 71(29.3%) received surgical operations, 183 (75.6%) received chemotherapies, and 110 (45.5%) received radiotherapies. The Kaplan–Meier analysis, log-rank test, and univariate Cox analysis showed that elder patients and patients with an advanced stage had a significantly shorter MST and a increased risk of death (*P*<0.05 in Table 4). But there were no significant effects of smoking status, histological subtype, surgical operation, chemotherapy, and radiotherapy on MST and death risk of male advanced NSCLC patients.

**Table 4:** Patient characteristics and clinical features.

Variables	Patients, n(%)(N = 242)	Deaths(n = 185)	MST (month)	Log-rank *P*	HR(95%CI)*
Age					
≤65	113(46.7)	83	16.5	0.034	Reference
>65	129(53.3)	102	11.7		1.37(1.02–1.83)
Smoking					
Never	17(7.0)	13	11.3		Reference
Former	142(58.7)	109	13.5	0.802	0.93(0.53–1.66)
Current	83(34.3)	63	13.4		0.85(0.47–1.56)
Histology					
Adenocarcinoma	81(33.5)	59	15.0		Reference
SCC	75(31.0)	55	15.8	0.060	0.87(0.60–1.26)
Others†	86(35.5)	71	10.8		1.31(0.93–1.85)
Stage					
IIIA	63(26.0)	42	18.9		Reference
IIIB	61(25.2)	44	14.7	0.001	1.25(0.82–1.91)
IV	118(48.8)	99	10.7		1.87(1.30–2.69)
Surgery					
No	171(70.7)	128	12.7	0.088	Reference
Yes	71(29.3)	57	15.0		0.76(0.56–1.04)
Chemotherapy					
No	59(24.4)	44	11.0	0.249	Reference
Yes	183(75.6)	141	13.7		0.82(0.58–1.15)
Radiotherapy					
No	132(54.5)	98	12.5	0.308	Reference
Yes	110(45.5)	87	15.0		0.86(0.64–1.15)

Abbreviations: MST, median survival time; SCC, Squamous cell carcinoma; HR, hazard ratio.

* Data were calculated by univariate cox regression analysis.

† Others include large cell, bronchioalveolar, mixed cell, undifferentiated and pathologic not otherwise specified carcinomas.

**Figure 1:**
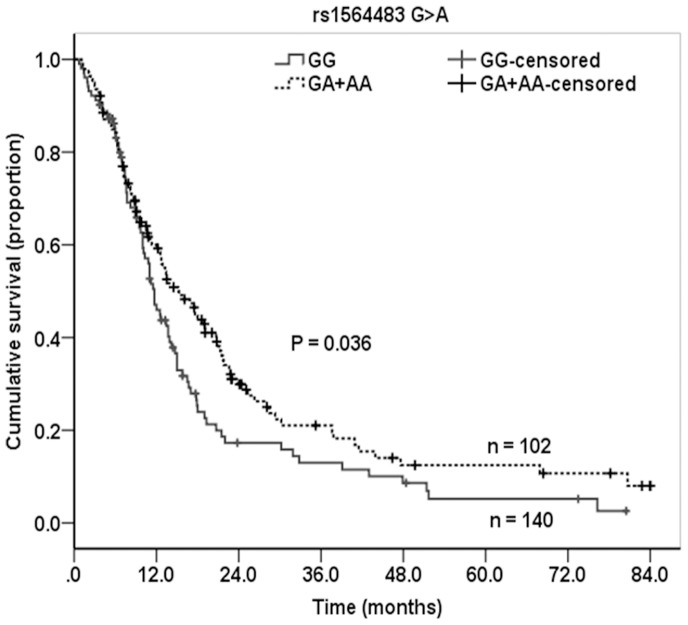
Kaplan-Meier survival curve for male advanced NSCLC patients by *BCL2* rs1564483 G>A genotypes.

As shown in Table 5, the Kaplan–Meier method and log-rank test showed that the male advanced patients carrying the *BCL2* rs1564483 GA and GA+AA genotypes had the MST of 16.9 and 15.2 months, which were significantly longer than the survival time of *BCL2* rs1564483 GG genotype carriers (MST = 11.7, Log-Rank *P = *0.025 and 0.036). The multivariate Cox regression models revealed that the adjusted hazard ratio (HR) for death was 0.69 for rs1564483GA, 0.66 for rs1564483AA, and 0.68 for rs1564483GA +AA genotype (Table 5, Figure 1), compared with the rs1564483 GG genotype. There was a dose-response effect of the rs1564483 G allele in reducing death risk (*P*_trend_ = 0.030). We did not observe a significant interaction of age or stage at diagnosis with *BCL2* rs1564483 GA+AA genotypes on the death risk of the study patients (*P = *0.413 and 0.802, respectively). For *BCL2* rs2279115 C>A and rs1801018 A>G polymorphisms, we did not find any associations of their genotypes with the survival outcomes of male advanced NSCLC patients.

**Table 5:** Genotype and haplotype analysis of *BCL2* with overall survival of male advance NSCLC patients.

Multivariables	Adjusted HR(95% CI)*	*P* *	Patients, n(%)	Death	MST (month)	Log-rank *P*
			N = 242	n = 185		
*BCL2_rs2279115*						
−938C/A						
CC	Reference	–	86(35.5)	68	13.4	–
CA	0.89(0.64–1.23)	0.477	115(47.5)	86	13.7	0.474
AA	0.96(0.62–1.49)	0.862	41(16.9)	31	11.5	0.796
*BCL2_rs1801018*						
+21A>G						
AA	Reference	–	209(86.4)	160	13.7	–
AG+GG	0.84(0.61–1.15)	0.633	33(13.6)	25	10.8	0.518
*BCL2_rs1564483*						
c*1204G>A						
GG	Reference	–	102(42.1)	83	11.7	–
GA	0.69(0.50–0.96)	0.027	103(42.6)	75	16.9	0.025
AA	0.66(0.42–1.05)	0.081	37(15.3)	27	12.9	0.374
GA+AA	0.68(0.51–0.93)	0.015	140(57.9)	102	15.2	0.036
	*P*_trend_ *	0.030				
*BCL2_*Haplotypes† (Frequency)						
C-A-G (44.0%)	Reference	–				
C-A-A (15.3%)	0.94(0.70–1.27)	0.702				
A-A-G (16.2%)	1.14(0.86–1.54)	0.358				
A-A-A (17.3%)	0.72(0.53–0.97)	0.033				

Abbreviations: MST, median survival time; HR, hazard ratio.

Note: The frequency of rs1564483GG genotype was 0.8% (2/242), so we combined rs1564483AG with GG genotype for analyses. Survival analyses were determined for haplotypes or diplotypes >5% frequency.

* Cox regression analysis was adjusted for age, smoking status, histology, TNM stage, surgery, chemotherapy, and radiotherapy status.

† Polymorphic bases were in the order of rs2279115 C>A, rs1801018A>G, rs1564483G>A from 5′ to 3′. Frequencies of haplotypes were determined using THESIAS v3.1 software.

### BCL2 Haplotypes and Survival of Male Advanced NSCLC Patients

When compared with the male advanced NSCLC patients with the *BCL2* CAG haplotype, there were no differences in death risk of patients carrying the CAA and AAG haplotypes (*P* = 0.702 and 0.358, respectively), but patients carrying the AAA haplotype had a significantly decreased death risk (HR = 0.71 and *P* = 0.033). In addition, compared with the *BCL2* AAG haplotype, the AAA haplotype was also associated with a significantly decreased death risk (HR = 0.62 and *P = *0.012, data not shown).

### Stratification Analysis for Associations between BCL2 rs1564483 and Overall Survival of Male Advance NSCLC Patients

The 242 male advanced NSCLC patients were further stratified by their features of age, smoking status, histology, TNM stage, and therapy treatments. We found that in the subgroups of smoking patients, stage IIIA patients, and patients underwent radiotherapy, the rs1564483 GA+AA genotype carriers have a significantly longer MST than the rs1564483 GG genotype carriers (Log-Rank *P = *0.037, 0.031, and 0.026, respectively) (Table 6). The association between rs1564483 GA+AA genotype and decreased death risk of NSCLC patients was more obvious in the smokers (HR = 0.68, *P = *0.016), stage IIIA patients (HR = 0.49, *P = *0.037), patients without surgery (HR = 0.67, *P = *0.047) but with chemotherapy (HR = 0.69, *P = *0.039) or radiotherapy (HR = 0.50, *P = *0.003) (Table 6). However, there were no significant interactions between the above characteristics and rs1564483 on the overall survival of NSCLC patients (all *P*_interaction_ >0.05).

**Table 6:** Stratification analysis for associations between *BCL2* rs1564483 (c*1204G>A) genotypes and overall survival of male advance NSCLC patients.

	GG		GA+AA					
Variables	n_p_/n_d_	MST	n_p_/n_d_	MST	Log- rank *P*	HR(95%CI)*	*P* *	*P*_interaction_ *
Age								0.690
≤65	43/34	12.0	70/49	17.6	0.187	0.68(0.42–1.09)	0.110	
>65	59/49	11.0	70/53	12.7	0.166	0.67(0.44–1.01)	0.057	
Smoking								0.900
Never-smokers	6/5	7.4	11/8	11.3	0.952	0.07(0.01–1.30)	0.074	
Smokers	96/78	11.7	129/94	15.3	0.037	0.68(0.50–0.93)	0.016	
Histology								0.549
Adenocarcinoma	31/24	11.5	50/35	17.4	0.514	0.73(0.39–1.34)	0.306	
SCC	34/27	15.8	41/28	17.6	0.146	0.74(0.41–1.35)	0.332	
Others^†^	37/32	9.7	49/39	12.6	0.138	0.68(0.41–1.10)	0.117	
Stage								0.340
IIIA	25/18	13.7	38/24	20.7	0.031	0.49(0.25–0.96)	0.037	
IIIB	29/24	15.8	32/20	12.5	0.828	1.16(0.60–2.24)	0.667	
IV	48/41	9.5	70/58	13.3	0.076	0.68(0.45–1.03)	0.069	
Surgery								0.953
No	71/55	11.7	100/73	13.4	0.117	0.67(0.44–0.99)	0.047	
Yes	31/28	11.7	40/29	17.4	0.147	0.57(0.32–1.02)	0.058	
Chemotherapy								0.551
No	34/26	10.0	25/18	11.3	0.401	0.57(.28–1.15)	0.117	
Yes	68/57	12.0	115/84	16.9	0.090	0.69(0.48–0.98)	0.039	
Radiotherapy								0.347
No	57/43	11.7	75/55	12.9	0.495	0.81(0.52–1.26)	0.349	
Yes	45/40	11.7	65/47	17.4	0.026	0.50(0.32–0.80)	0.003	

Note: n_p_/n_d_, number of all patients/number of death patients; SCC, Squamous cell carcinoma.

* Data were calculated by Cox regression analysis, adjusted for age, smoking status, histology, TNM stage, surgery, chemotherapy, and radiotherapy status. The GG genotype was used as the reference group.

## Discussion

To our knowledge, this is the first study to provide evidence that genetic variations of *BCL2* may play an important role in predicting the susceptibility of lung cancer and overall survival of advanced NSCLC patients in male Chinese. We found that the *BCL2* 3′-UTR rs1564483 A allele has a inverse dose-response relationship with lung cancer risk, which was more evident in the elders, smokers, and subjects without family history of cancer. Furthermore, the *BCL2* 3′-UTR rs1564483 A allele was associated with a favorable survival outcome for male advanced NSCLC patients, and this effect was more obvious in patients with smoking, stage IIIA, and in patients without surgery but underwent chemotherapy or radiotherapy.

Bcl-2 is one of the most important proto-oncogene that can promote tumorigenesis through inhibiting intrinsic apoptotic pathway [5]. The *BCL2* gene was first discovered at the t(14,18) chromosome translocation breakpoint in B-cell follicular lymphomas, and the previous literatures mainly focused on the function and association of *BCL2* variants with risk of leukemia [16], [17]. Studies of Bcl2 were also carried out in the solid tumors, including lung cancer. Bcl-2 is expressed relatively early during bronchial preneoplasia [18]; both small cell lung cancer (SCLC) and NSCLC showed over-expression of Bcl-2 protein [19], [20]. The anti-apoptotic function of Bcl-2 is closely associated with its expression levels [6]. Until recently, only one Caucasian study suggested that the *BCL2* rs1462129 C and rs2551402 A allele were associated with increased lung cancer risk (661 Cases and 959 controls), but these associations were not replicated in a larger Caucasian population of 1154 lung cancer cases and 1073 controls, although the *P*-value in the result of pooled dataset reached the significance level. In addition, these two SNPs (rs1462129 and rs2551402) are located in the intron and non-regulated region of *BCL2* gene, so they may not possible be the causal variants [11]. The expression of Bcl-2 can be regulated at both the transcriptional and post-transcriptional levels. An important mechanism of the latter modification is based on the *BCL2* mRNA stability, which is mainly controlled by the 3′-UTR of *BCL2* gene [21]. Many miRNAs, including miR-181b [22], miR-200bc/429 [23], and miR-204 [24], have been reported to bind to the *BCL2* 3′-UTR and modulate *BCL2* mRNA levels. In our study, we found that the rs1564483A allele in 3′-UTR of *BCL2* gene was associated with reduced lung cancer risk in the male Chinese, and this effect was more evident in the elders, smokers, and subjects without family history of cancer. One can suspect that the rs1564483 G to A substitution might change the stem-loop structure of 3′-UTR or introduce a miRNA binding site, which may affect Bcl-2 mRNA stability and expression levels. The decreased Bcl-2 will reduce anti-apoptosis ability of normal lung cells, and then contribute to a protection role against carcinogenesis. However, the real underline mechanism warrants further investigation.

In most lung cancers, one main mechanism of cancer cell resistant is the so-called non-pump drug resistance that is primarily due to the activation of anti-apoptotic cellular defense of lung cancer cells, and Bcl-2 is thought to be a key player in this defense [10], [25]. Bcl-2 protein may exert distinct biological effects in different cell types [26]. Previous studies showed that *BCL2* −938CC (rs2279115) genotype were associated with poor prognosis for renal cancer [27] for SCLC patients [28], but with favorable survival for patients with chronic lymphocytic leukemia [29] and B large lymphoma [30], while the +21AA (rs1801018) genotype had a significantly longer survival time in acute myeloid leukemia [31]. However, the above studies did not explore the rs1564483G>A polymorphism in their survival analyzes and no studies have investigated the associations of *BCL2* variants with the prognosis of NSCLC cancer patients yet. In our study, we found that the *BCL2* rs1564483A allele was associated with a significantly favorable survival of male advanced NSCLC patients, especially in smoking patients and patients underwent chemo- or radio-therapy. If patients with rs1564483 A allele were associated with a lower level of Bcl-2 protein, it may be biologically plausible that the decreased anti-apoptotic cellular defense of lung cancer cells may improve the efficacy of chemo- or radio-therapy strategies, and then lead to a favorable outcome of advanced NSCLC patients.

Despite extensive effort has been made during the past three decays, the treatment outcomes of NSCLC patients, especially of the advanced NSCLC patients NSCLC, are yet to be considered dismal [32]. Genetic profile based molecular targeted therapy has become one of the most promising approaches for improving the individual’s prognosis of cancer patients. Several randomized Phase III trials have found that combining bcl-2 antisense with chemotherapy can improve antitumor response, increase apoptosis of tumor cells, and increase survival of NSCLC patients [20], [33]. We can expect that combined analyses of the *BCL2* polymorphisms and patients’ clinicopathologic features may help predict the survival outcomes of NSCLC patients.

Emerging evidence demonstrated that there were very clear differences in biology, histological subtypes, susceptibility, and response to therapy between lung cancer in men and women [34]–[36]. The epidemiological studies of lung cancer with imbalances in terms of sex may bias the results and lead to bias conclusions. In this study, in order to avoid the gender disparity, we focus on the male subjects to explore the associations of *BCL2* variants with male Chinese. This study also has some limitations. For example, since all subjects enrolled in this study were ethnic Han Chinese and the biological function of rs1564483 G>A polymorphism remained unclear, additional studies are needed to validate the associations in the other human races and uncover the biological function of rs1564483 G>A polymorphism in regulation of Bcl-2 expression.

In conclusion, Our study provide the first evidence and preliminary findings that the rs1564483 A allele located in the 3′-UTR of *BCL2* gene was associated with a significantly lower risk of lung cancer in male Chinese and with a favorable prognosis of advanced NSCLC males. However, these findings need to be validated by additional population-based prospective studies as well as uniformed clinical trials, and the potential molecular mechanisms of rs1564438G>A polymorphism need elucidation by further biological studies.
